# Supplementary material for: The β-NGF/TrkA Signalling Pathway Is Associated With the Production of Anti-Nucleoprotein IgG in Convalescent COVID-19
Source: Front Immunol. 2022 Jan 14;12:813300. doi: 10.3389/fimmu.2021.813300 (PMC8795736; doi:10.3389/fimmu.2021.813300)
Supplement: Supplementary file 1 [file DataSheet_1.docx]

Supplementary Material

# Supplementary Table 1. Demographic characteristics of the 40 selected participants; p-values lower than 0.05 were considered significant. ns= not significant

| **PCR** | **Anti-NP** | **N** | **Ethnic composition (Asian; Black; White)** | **Age (median; IQR)** | **% of females** |
| --- | --- | --- | --- | --- | --- |
| - | - | 10 | 4; 0; 6 | 37; 42.25-30 | 50 |
| + | + | 10 | 3; 0; 7 | 38; 51.5-29.25 | 50 |
| + | - | 7 | 4; 0; 3 | 34; 36.5-27.5 | 42.9 |
| N/A | + | 13 | 3; 1; 9 | 40; 48-34 | 61.54 |
| p-value | | | | ns | ns |

#
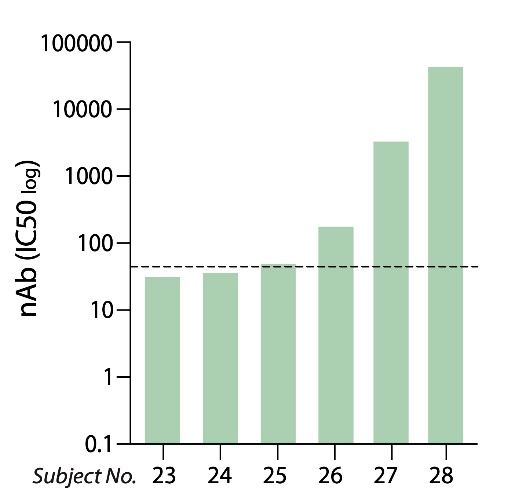
Supplementary Figures


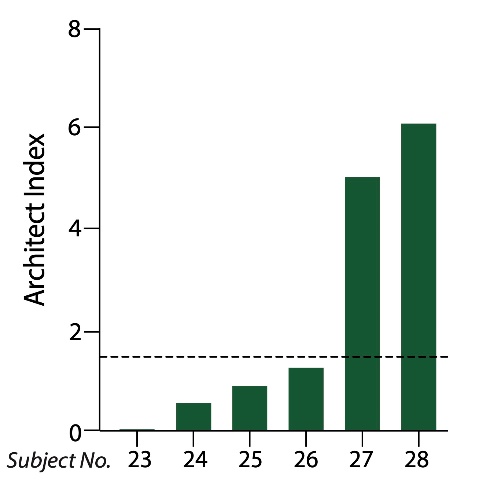


C

B

A


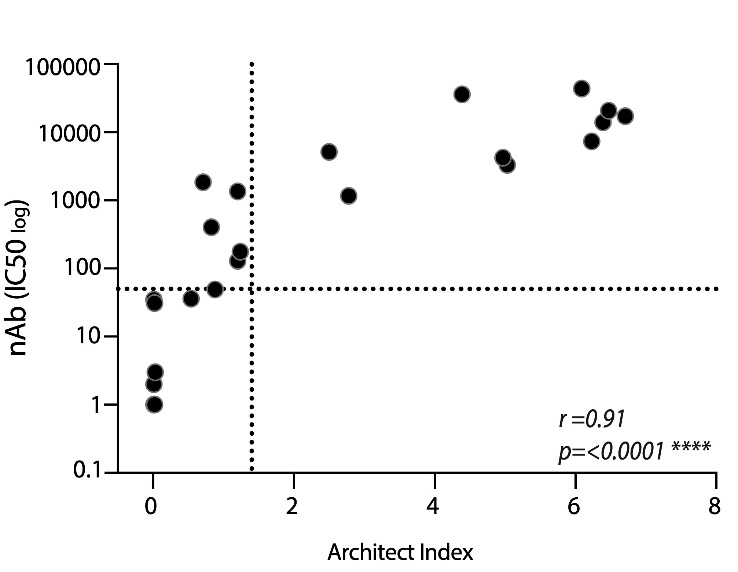


**Supplementary Figure 1.** Comparison between anti-NP levels and nAb titres. (A) anti-NP IgG levels expressed as Architect Index (manufacturer arbitrary units) for subject analysed in the study for which T cell ELISpot analysis was not undertaken (ordered lowest to highest); the dotted line represents the 1.4 cut-off, below which samples are considered as negatives. (B) Neutralising antibody (nAb) titres (IC50) corresponding to the same subjects in (A); the dotted line represents the cut-off below which samples are considered as negatives (IC50=50). (C) Correlation between anti-NP IgG levels and nAb titres in all subjects (n=28). The dotted lines represent the cut-offs below which samples are considered negatives for each assay. A Spearman non-parametric correlation test was used to determine significance, *p<0.05; **p<0.01; ***p<0.001, ****p<0.0001, ns = not significant.

**
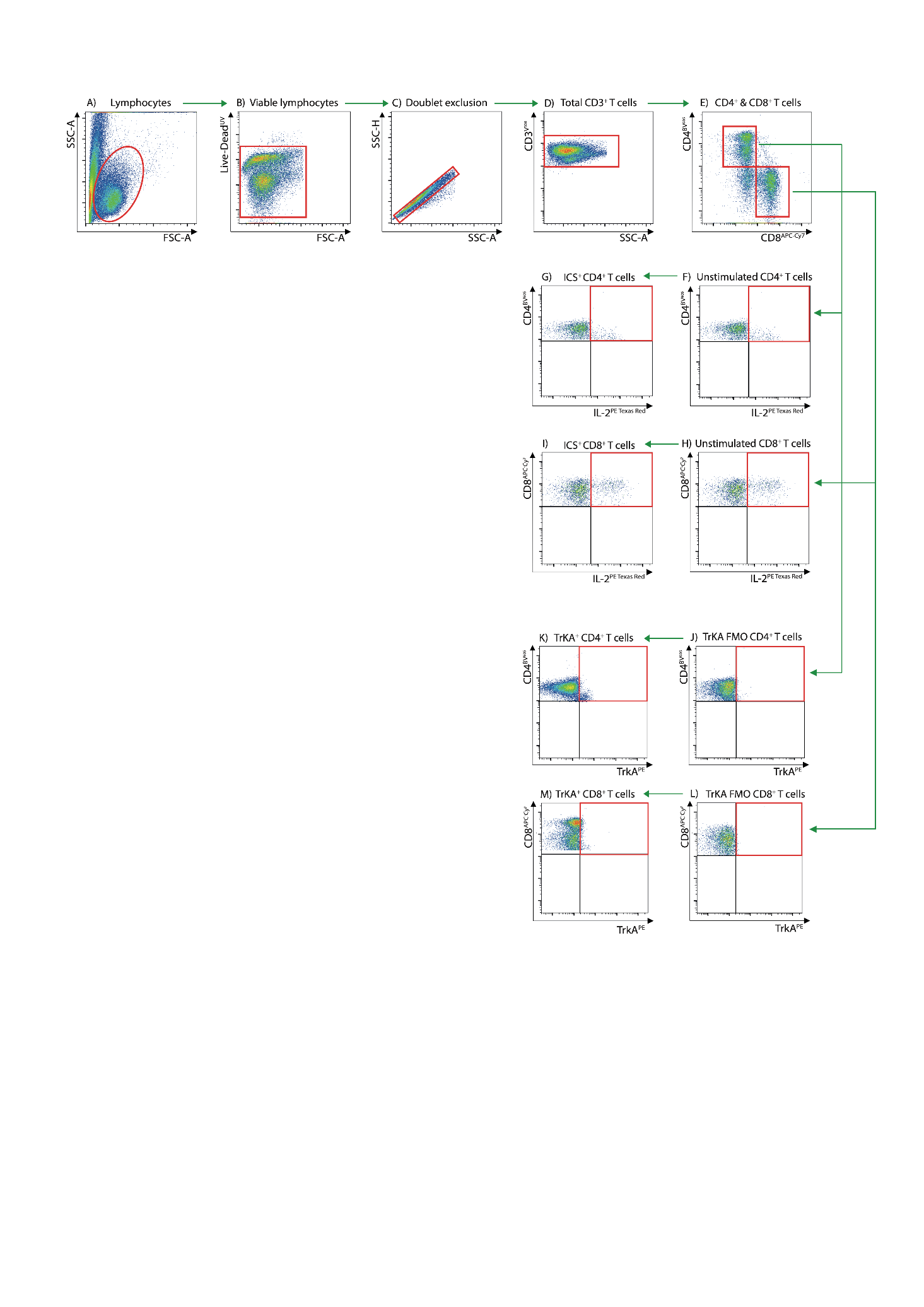
Supplementary Figure 2.** Flow cytometry gating strategy. Initial gating of (A) Total lymphocytes with forward scatter (FSC)/side scatter (SSC). To include only (B) viable cells for analysis, dead cells were excluded using a viability dye. (C) Single cells were then identified defined on an SSC-A/SSC-H plot. (D) Cells were then gated on the CD3+ population, and further gated on the (E) CD4+ and CD8+ subpopulations. Antigen-specific CD4+ and CD8+ cells were gated according to the cytokines produced, (F) unstimulated CD4+ T cells and (G) intracellular cytokine staining (ICS) staining of CD4+ T cells following peptide stimulation (IL-2 shown), in addition (H) unstimulated CD8+ T cells and (I) ICS staining of CD8+ T cells following peptide stimulation (IL-2 shown). TrkA analysis, by (J) FMO on CD4+ T cells and (K) with TrkA strained CD4+ T cells, in addition (L) FMO on CD8+ T cells and (M) with TrkA strained CD8+ T cells.


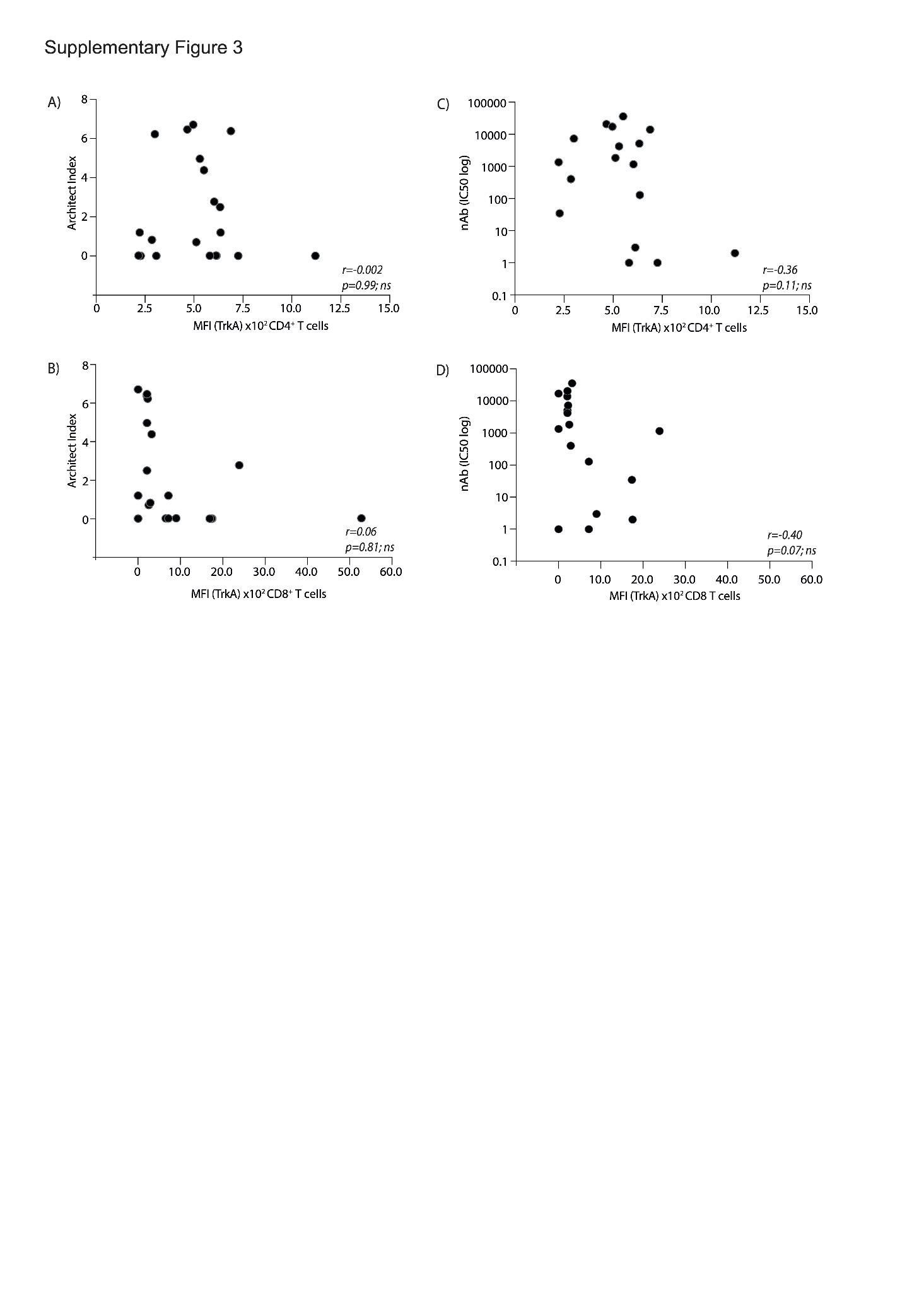


**Supplementary Figure 3.** TrKA+ T cells in relation to antibody production. Correlative expression of anti-NP levels (Architect index) in relation to TrKA+ expressing (A) CD4+ and (B) CD8+ T cells along with nAb levels in relation to TrKA+ expressing (C) CD4+ and (D) CD8+ T cells. Spearman non-parametric correlation tests were used to determine significance, *p<0.05; **p<0.01; ***p<0.001, ****p<0.0001, ns = not significant.
